# Adhesives for Achieving Durable Bonds with Acetylated Wood

**DOI:** 10.3390/polym9120731

**Published:** 2017-12-20

**Authors:** Charles R. Frihart, Rishawn Brandon, James F. Beecher, Rebecca E. Ibach

**Affiliations:** USDA Forest Service Forest Products Laboratory, Madison, WI 53726-2398, USA; rbrandon@fs.fed.us (R.B.); jbeecher@fs.fed.us (J.F.B.); ribach@fs.fed.us (R.E.I.)

**Keywords:** wood, adhesion, acetylated, planing, hydroxyls, phenolic, epoxy, isocyanate, strength, failure

## Abstract

Acetylation of wood imparts moisture durability, decay resistance, and dimensional stability to wood; however, making durable adhesive bonds with acetylated wood can be more difficult than with unmodified wood. The usual explanation is that the acetylated surface has fewer hydroxyl groups, resulting in a harder-to-wet surface and in fewer hydrogen bonds between wood and adhesive. This concept was evaluated using four different adhesives (resorcinol–formaldehyde, emulsion polymer isocyanate, epoxy, and melamine–formaldehyde) with unmodified wood, acetylated wood, and acetylated wood that had been planed. Strikingly, acetylation did not hinder adhesive bonds with a waterborne resorcinol–formaldehyde adhesive that bonded equally well to both unmodified and acetylated yellow poplar. An epoxy adhesive bonded better to the acetylated wood than to the unmodified wood, in contrast to an emulsion polymer isocyanate, which gave less durable bonds to acetylated than to unmodified wood. Planing of the acetylated wood surface prior to bonding reduced bond durability for the epoxy adhesive and increased the amount of surface hydroxyl groups, as measured using X-ray photoelectron spectroscopic analysis of the trifluoroacetic anhydride-treated wood. These experiments showed that wood modification is useful in understanding wood-adhesive interactions, in addition to determining how to develop adhesives for acetylated woods.

## 1. Introduction

Two major disadvantages of using wood for structures are that it shrinks and swells as the moisture changes and it can be degraded by biological means [1]. Preservative treatments with some inorganic and organic chemicals improve decay resistance, but most of these do not improve dimensional stability and add potential environmental or health risks [2]. Alternatively, thermally and chemically modifying the hydroxyls of wood can make it more dimensionally stable [2,3]. Acetylation is one chemical modification that not only reduces decay, but also provides good dimensional stability. These beneficial properties have led to the commercial production of Accoya^®^ wood. Although acetylated wood provides durable wood products, forming a durable adhesive bond with some types of adhesives can be more difficult for acetylated wood than for unmodified wood [4]. In general, good results have been obtained with phenolic and polyurethane adhesives [4,5], but a better understanding of factors that control the durability of adhesive bonds to acetylated wood not only is of commercial importance but also helps to comprehend wood adhesion in general.

The first step in forming an adhesive bond is for the adhesive to wet the substrate surfaces (both applied and transfer) by flowing over them and into any pits or crevices on their surfaces. Without molecular level contact and some level of adhesion, the only interaction is mechanical interlocking. Surface wetting and adhesion are a problem for many adhesive applications because of the low surface energy for either the substrate or the chemicals absorbed on the substrate’s surface. Waterborne adhesives work well for bonding freshly prepared wood surfaces. In addition, the cellular structure of wood provides a large bonding surface area. The second step for bonding is for the adhesive to form interfacial interactions between the adhesive and the substrate. The large number of hydroxyl groups on the wood polymers has been thought to result in many hydrogen bonds between the adhesive and the wood surface, providing strong, durable bonds. Both the wetting and adhesion can be hindered by modifications of the wood surface.

The literature has generally concluded that the conversion of hydroxyl groups to acetyl groups should hinder both the wetting and the hydrogen bond formation processes. An early study on bonding acetylated strands showed difficulty in forming good bonds [6]. However, a later study to improve adhesion by using surfactants did not improve adhesion, showing that the issue is more complicated than just surface polarity [7]. The concept that acetylation makes surfaces harder to wet and bond has continued to be cited without convincing data showing that lower surface adhesion was the cause of bond failure.

This work builds on a prior publication [4], with the goal of better understanding the factors controlling the durability of wood bonds. This prior study involved 18 adhesives with four degrees of acetylation (0, 8, 14, and 20 weight percent gains) of yellow poplar (*Liriodendron tulipifera*). These thermoplastic and thermosetting adhesives included emulsion polymer isocyanates (EPI), polyurethane, moisture-curing hot-melt, poly(vinyl acetate)s, neoprene and waterborne contacts, casein, epoxy, melamine–formaldehydes (MF), urea–formaldehydes, resorcinol, resorcinol–phenolics, (PRFs), and phenolics. From these 18 adhesives, we selected four that seemed to represent different cases. The resorcinol-formaldehyde (RF) adhesive provided durable bonds independent of whether the wood was acetylated or not. The MF adhesive lost both dry and wet strength adhesion as the degree of acetylation increased. An emulsion polymer isocyanate adhesive maintained dry strength, but lost wet strength adhesion as the degree of acetylation increased. The epoxy–polyamide adhesive lost some dry strength with acetylation, but regardless had poor bond strength under wet conditions. A follow-up study used select wood species with the heartwood and sapwood of Scandinavian pine and Norway spruce with phenolics, cross-linked poly(vinyl acetate) (PVAx), and EPI [8]. This research tested bonding of laminates with different levels of acetylation to form glulams and testing in a more severe delamination test. Again, the adhesive with the most durable bonds was the RF, with the PRF nearly as good. The EPI did well on both unmodified and acetylated wood only under dry conditions. The PVAx did much better on acetylated wood, which was attributed to the lack of swelling with the acetylated wood.

These varied responses of different adhesives to acetylation and exposure to water soaking provide interesting information on factors controlling bond strength [4]. Although detailed discussion was provided of each adhesive and of the overall effect, there are different ways to interpret the data. An important aspect of moisture-related bond durability is that the in situ polymerized adhesives interact differently from the prepolymerized adhesives during wetting and drying [9]. This is in addition to the effect of interactions of specific adhesives with the wood as the acetylation content increases. Another factor is that acetylated wood maintains higher strength when water soaking due to lower water uptake and less plasticization [2,3,10]. All these factors must be taken into consideration when interpreting the results.

The prior study [4] also considered the effect of acetylated wood being planed prior to bonding; planing of the surface is a standard procedure in structural wood bonding to provide a flat, smooth, fresh, and active bonding surface. However, bonds in composites are formed from unplaned acetylated surfaces and can be represented by unplaned acetylated wood. Thus, we examined both unplaned and planed acetylated wood for its bondability and its surface hydroxyl contents. For knowing the latter, we developed a procedure to measure surface hydroxyl contents using a treatment with trifluoroacetic anhydride, followed by X-ray photoelectron spectroscopy (XPS) to measure surface hydroxyl contents.

This research involved the use of four adhesives (RF, EPI, Epoxy, and MF) with unacetylated and acetylated wood (both planed and unplaned), measuring dry and wet strengths, and then carefully examining the failure surfaces.

## 2. Materials and Methods

### 2.1. Bond Strength Study

#### 2.1.1. Experimental Design

The experiment was designed to survey an array of thermosetting adhesives for their ability to bond to planed and unplaned acetylated wood. Effectiveness of bonds was determined according to ASTM D905 [11] by measuring shear strength and wood failure in an ambient (dry) condition and in a wet condition after water soaking under vacuum and then pressure (VPS), keeping the samples soaking until being tested [12].

The design was a full factorial arrangement with four adhesives (PF, MF, EPI and epoxy) and three treatments (acetylated planed, acetylated unplaned, and an unacetylated, planed control) yielding 12 treatment combinations. Each treatment combination (wood modification and adhesive used) was replicated nine times, and four samples were taken from each replicate. After randomly assigning the specimens for dry or wet tests, the 432 samples (four adhesives, three wood modifications, four samples from each of the nine specimens) were tested for their shear strength and wood failure in dry and wet test conditions.

#### 2.1.2. Acetylation

Yellow-poplar (*Liriodendron tulipifera*) sapwood lumber, free of defects, was sawn into strips 31.8 mm wide, 229 mm long, and thick enough so that they could be mechanically planed to 6.4 or 7.9 mm thick, (see bonding section). After cutting, the strips were placed in an oven and dried at 105 °C for 24 h. The strips were removed from the oven, cooled in a desiccator for 1 h, and weighed.

The strips were acetylated according to the following procedure. Strips were placed in a 2 L glass reactor fitted with a reflux condenser. The glass reactor was filled with enough acetic anhydride to cover the strips even after absorption of chemical. The acetic anhydride and wood were heated to boiling (139.8 °C) for 4 h and then cooled to room temperature (21 °C). Strips were removed, washed for 4 h in reversed osmosis water to remove acetic acid and excess acetic anhydride, air dried overnight, and then oven-dried for 24 h at 105 °C. Weight gain due to acetylation was determined after oven-drying by calculation as a percentage of the original oven-dried weight. The acetylated wood strips had an average of 21.7 ± 0.9 weight percent gain.

#### 2.1.3. Bonding

All strips, including the unmodified controls, were conditioned at 27 °C and 65% relative humidity until bonded. If the strips were to be planed, they were 7.9 mm thick and were planed after treatment and before bonding. If the strips were not to be planed after acetylation, they were planed to 6.4 mm thickness prior to treatment. Specimens were prepared by laminating two strips of wood, 6.4 mm thick, 31.8 mm wide, and 229 mm long. Adhesive was placed on one piece of wood on a balance and rolled out for even coverage and desired adhesive weight.

Cold-setting adhesives, RF: Casophen resorcinol–formaldehyde RS-216 (five parts) with FM-60M catalyst (one part) (Borden (Hexion), Bellevue, WA, USA) with 15 min open and 25 min closed time, EPI: Isoset emulsion polymer isocyanate WDC-154 (100 parts) with CX-11 catalyst (17 parts) (Ashland, Dublin, OH, USA) with 15 min open and closed assembly time, and Lord 305-1 epoxy (two parts) with 305-2 hardener (one part) (Lord Corporation, Cary, NC, USA) with one h closed assembly time, were spread at an approximate rate of 320–340 g/m^2^ and cured at room temperature. Adhesive was spread on both surfaces with a rubber-roll hand spreader. Adhesive spread rate was accurately controlled by automatically tare-weighing the adhesive on the laminates as they were spread. Pressure for the epoxy and emulsion polymer isocyanate was determined by increasing pressure until the beginning of squeeze out. After a 15-min open assembly time and 25-min closed time, pressure for the resorcinol–formaldehyde was maintained at 690 ± 35 kPa for 18 h. All nine replicates (joint assemblies) of a single treatment combination were pressed within the same press closure. Closed assembly varied between 15 and 60 min, depending on individual curing characteristics.

The hot setting melamine-formaldehyde (MF), Cascomel MF-600 (Borden (Hexion)) was spread at a rate of 200 kg/m^2^ with 35 min open time and 25 min closed assembly time and was cured in an electrically heated laboratory hot-press maintained at 138 ± 5 °C. Pressure was maintained at 862 ± 35 kPa for 10 min.

After removing material from both sides and ends, four block-shear samples with a shear area of 2.54 by 2.54 cm were cut from each specimen to form shear blocks as described in ASTM D905 and randomly assigned to either the dry or wet shear tests.

#### 2.1.4. Adhesive Testing

Eighteen samples representative of each wood modification and adhesive used treatment combination (216 total) were subjected to a single vacuum pressure soak (VPS) and then tested for shear strength and wood failure while in the water-saturated condition. The saturation process consisted of the following events:

1. Submerged specimens in tap water at room temperature in a pressure vessel.
2. Maintained a vacuum of 635 ± 85 kPa for 30 min.
3. Maintained a pressure of 448 ± 35 kPa for 30 min.
4. Remained submerged in water until tested.

Dry and wet samples were tested in a compression-loading shearing tool as described in ASTM Method D905 [11] using a MTS 810 Material Test Machine (MTS Systems Corporation, Eden Prairie, MN, USA). Load was applied at a constant rate of 2.54 mm per minute until failure. The maximum load at failure was recorded, and then shear strength was calculated for each specimen based on the shear area. Wood failure was estimated to the nearest 5% on the sheared area, according to ASTM D5266-99 [12]. The wet-tested samples were air-dried before estimating wood failure [13]. Estimating is easier after drying because of greater color and light reflection contrast between the dry wood fiber and the adhesive.

#### 2.1.5. Shear Data Analysis

The shear test and wood failure results are presented using the standard (one standard deviation) error bars. As indicated in the Supplementary Materials, further data analysis was performed using analysis of variance (ANOVA) for shear strength and wood failure of all four adhesives under dry and wet conditions using the generalized linear model (GLM) procedure of the SAS Institute Inc. (Cary, NC, USA) system.

### 2.2. Analysis of Hydroxyl Groups on Wood Surface

#### 2.2.1. Treatment with Trifluoroacetic Anhydride

Portions from the outer surface of the acetylated and unmodified yellow-poplar specimens were isolated. These portions were about 1 mm thick and about 1 cm square to reduce the volume to facilitate drying. Some of these specimens were treated with trifluoroacetic anhydride (TFAA) using the following method that was developed using paper specimens to validate the methodology [14]. Wood specimen squares (~1.2 by 1.2 cm) were placed on aluminum foil in a 50-mL weighing bottle along with a 2-mL beaker containing 0.5 mL TFAA. These were allowed to react for 1 h at room temperature in the closed bottle. The weighing bottle was then partly opened in a fume hood to vent the volatiles (TFA and its anhydride). The TFAA-treated wood was placed in folds of aluminum foil and dried by evacuating overnight at 5–10 Pa.

#### 2.2.2. X-ray Photoelectron Analysis

X-ray photoelectron spectra were obtained using the Perkin Elmer 5400 ESCA spectrometer (Perkin Elmer, Wellesley, MA, USA) at the University of Wisconsin’s Material Science Center in Madison, Wisconsin. This instrument was equipped with a twin anode X-ray source and used a hemispherical electron energy analyzer. Small specimens (1 cm^2^) (approximately 1 mm in thickness) were mounted on a stainless steel stub using double-coated conductive tape [14]. The XPS was conducted in an ultra-high vacuum environment (approximately 10^−6^ Pa). The specimens were dried overnight in a vacuum chamber pumped by a turbo-molecular vacuum pump to remove the water from the wood, then further degassed for 20–30 min in the specimen antechamber before analysis.

Spectra were obtained using a Mg K-alpha X-ray source energized to 300 W and a pass energy of 35 eV for the electron analyzer. The X-ray source window was approximately 1 cm away from the specimen surface. Changes in source specimen distance, orientation of specimen with the grain parallel or perpendicular to the source, or source power showed no effect as determined by the emission curve on wood, epoxy, or paper. However, fluorocarbons are known and observed to degrade under this condition because of exposure to electrons generated by the X-rays passing through the aluminum window of the X-ray gun [15]; thus, the exposure times were kept short and the source power was low.

#### 2.2.3. Curve Fitting

The data from each of the high-resolution spectra were manipulated in the same way as for the preliminary study with paper [14] using AugerScan software (RBD Enterprises, Bend, OR, USA). First, contributions from the electron emission stimulated by the 3, 4 components of the Mg K-alpha X-ray emission were subtracted. Points were manually selected to represent a baseline, and a nonlinear background was subtracted from each spectrum, according to conventional practices. Each of the high-resolution spectra was fitted with Gaussian–Lorentzian components to characterize the surface chemistry, with important details, including the use of cellulose as a control for determining degree of acetylation on the surface, given in a prior publication [14]. Spectra were obtained from oxygen 1s electrons and fluorine 1s electrons when these atoms were present. Only carbon 1s spectra, however, were used to quantify the surface acetylation chemistry. There was sufficient resolution to determine the needed information and the method has two advantages: (1) no need to be concerned with relative response of the electron multiplier to electrons of different energy (electrons from fluorine or oxygen, etc.); and (2) no need to be concerned that spectra of different elements sample different depths of material. A scaled portion of the spectrum was fitted to each specimen spectrum to account for artifact contributions.

## 3. Results

Acetylation of wood has been used to selectively modify the hydroxyl groups in wood for greater dimensional stability and decay protection, and the procedures are well established [2,3,10]. Immersion of the wood in acetic anhydride under reflux conditions was used in these experiments to achieve high acetylation. The weight gain was calculated by comparison of the dried sample before and after modification and in this experiment was 21.7 ± 0.9%, which is a typical value for a nearly complete acetylation of wood.

### 3.1. Surface Treatment and Analysis

For bonding of wood, the wood is typically planed or sanded to provide a fresh and more activated surface; this is important because the surface energy of wood declines rapidly after preparation [16]. In the prior study on wood bonding by Vick and Rowell [4], not only was the unmodified wood planed, but so was the acetylated wood [4,17]. This planing for the acetylated wood could expose previously sterically unreachable (not acetylated) hydroxyl groups. An important research question was whether this planing caused the surface to be more polar than a completely acetylated one. Both ways are legitimate approaches in that planing represents how glulams and similar products are made commercially, but planing the wood after the veneer, strands, particles are acetylated would not represent how panel products (such as plywood, oriented strandboard, particleboard, and fiberboard) are produced. Important elements of this program were to understand how many surface hydroxyl groups are exposed when the acetylated wood is planed and how planing influences the bondability of acetylated wood.

To address the first element, we needed to measure accessible hydroxyl groups on the surface of the wood. Because hydroxyl groups are difficult to quantify by XPS, trifluoroacetic anhydride (TFAA) was used to label squares (~1.2 by 1.2 cm), which were cut from the unmodified and acetylated wood (both planed and unplaned) [14]. Vapor phase treatment with TFAA gave a similar degree of reaction with surface hydroxyl groups as did the liquid TFAA treatment [14]. One of the advantages of using TFAA vapor to label hydroxyl groups by this method is that no liquid exposure of the wood is necessary, and the samples are not saturated with excess reactants that would need to be removed [18]. The TFAA is very volatile, with a boiling point of 62 °C. The reaction product trifluoroacetic acid (boiling point 72 °C) is also volatile, in contrast to 139 °C for acetic anhydride and 118 °C for acetic acid. Thus, the excess materials can be removed without washing, which is often used for the acetylation.

**Figure 1:**
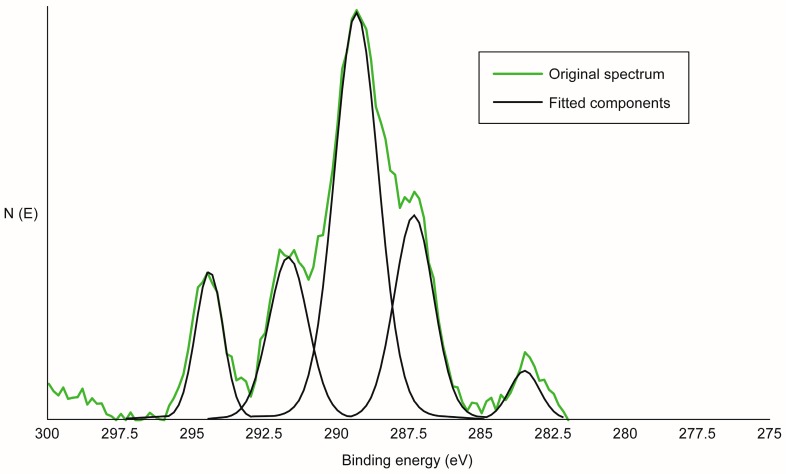
Carbon 1s spectrum of yellow-poplar treated with trifluoroacetic anhydride (TFA); the number of electrons measured (N (E)) plotted vs. binding energy (eV) [14].

Next, a method was developed that minimized any contamination in sample handling and X-ray photoelectron spectroscopy (XPS) analysis of the sample [14]. XPS is a useful tool for surface analysis because it is surface sensitive, identifies nearly all elements, and frequently discriminates between different bonding states. The trifluoroacetylated versions of the unmodified wood and acetylated (both planed and unplaned) wood were used in developing the detailed methodology for obtaining and analyzing the data. Once the data were obtained, the overlapping signals needed to be deconvolved to obtain the relative amounts for each bonding state. Figure 1 shows the carbon 1s spectrum of yellow-poplar treated with TFA showing the original spectrum and the fitted peaks, with full details provide in reference [14]. The fluorine causes a shift in the acetylated portion to higher binding energies than does the hydrogen atoms. Thus, we can measure the degree of acetylation versus trifluoroacetylation by XPS.

The data in Table 1 show that planing the acetylated wood surface led to an increase in TFAA reactive sites from 9% of the originally available hydroxyl sites to 42%. Not all hydroxyls near the surface are hydroxylated in acetylated wood, but the act of planing dramatically increases the number of free hydroxyl groups.

On the basis of the relative amounts of free OH groups, it is likely that the explanation of poor bonding due to a much less polar surface after acetylation and planing seems more doubtful. In addition, the data support the need for this study to determine the comparison of unmodified wood with acetylated wood with and without planing.

We tried to acetylate just the wood surface by using acetic anhydride vapor treatment but were unable to find the right conditions because the vapors went too deeply into the wood. The depth of acetylation was determined by taking microtome layers from the surface and measuring the degree of acetylation by aqueous sodium hydroxide hydrolysis under heat using propionic acid as an internal standard. The acetate content of the diluted reaction mixtures was determined by anion exchange chromatography (AEC) with suppressed conductivity detection [19]. Therefore, we did not test the performance of wood with just the surface acetylated.

### 3.2. Bonding Studies

Results from the surface analysis of planed acetylated wood showed that bonding should also include the unplaned acetylated wood as well as the unmodified wood. Using conditions and adhesives from the prior study [4] allowed obtaining additional data to help interpret the results. The prime questions were how much the acetylation influenced the bond strength of wood assemblies and how much the planing of the acetylated wood influence the bond strength of the acetylated wood assemblies. Performance of these bonded assemblies was determined under ambient conditions (dry) and after vacuum pressure water soaks (wet) using ASM D905 specimens [11].

**Figure 2:**
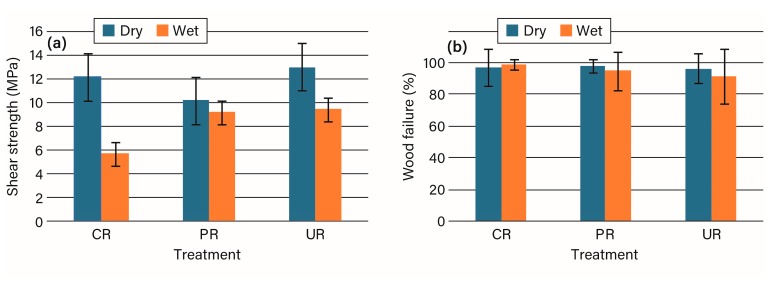
The (**a**) shear strength and (**b**) percentage wood failure for resorcinol-formaldehyde bonding of unmodified (CR), planed acetylated (PR), and unplaned acetylated (UR) wood.

Resorcinol adhesives, along with most phenolics, are used for structural and exterior bonded wood products because they provide the most durable and heat-resistant bonds. A general assumption could be that waterborne phenolics would not provide good bonds because of an inability to wet and bond to the hydrophobic acetylated wood surface. Some data tended to support this thought [6], while other data did not [7,8]. The data in Figure 2a, and Tables S2 and S4 show good dry strength for all specimens and good wet strength except for the unmodified wood, which is similar to the data from an earlier study [4]. Given that the amount of wood failure is high in all cases, these tests are really measuring wood strength. As is well known, wood becomes plasticized by water, which results in a large strength decrease for the unmodified wood compared to the acetylated wood for the wet tests with the acetylated wood not absorbing much water. This points out one difficulty in bonding acetylated wood, which is not often recognized: for wet tests, the acetylated wood is going to apply a much higher force on the bondline than does the unmodified wood. Planing the acetylated wood affected the dry wood specimens more than the wet wood specimens, which may be due to the more brittle nature of the acetylated wood that we observed in planing acetylated wood. Although the acetylated wood did not absorb much water, it did not seem to interfere with strength development for the RF adhesive, see Figure 2, and Tables S3 and S5. In addition the polar RF developed good bond strength with the low polarity acetylated wood.

**Figure 3:**
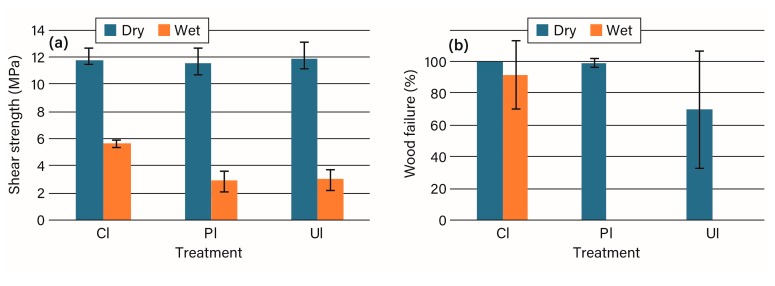
The (**a**) shear strength and (**b**) percentage wood failure for emulsion polymer isocyanate bonding of unmodified (CI), planed acetylated (PI), and unplaned acetylated (UI) wood.

The emulsion polymer isocyanate (EPI) was chosen because it had good dry shear strengths and wood failure on both unmodified and acetylated wood, but it had lower strength and wood failure on wet tests with the acetylated wood [4]. The results in Figure 3, Tables S2 and S4 are similar in that dry strengths and wood failure all remained high with acetylated wood. There was some drop in dry wood failure for the unplaned acetylated wood, which was not tested in the prior program, but there is also a wide scatter in the data. The wet tests showed a drop in wood failure similar to the prior study, but wet shear strength showed a larger decrease with acetylated wood in contrast to the prior study Figure 3. The potentially slower water removal rate [10,20] may cause more of the isocyanate to be consumed in reaction with the water than developing structure with the polyol.

**Figure 4:**
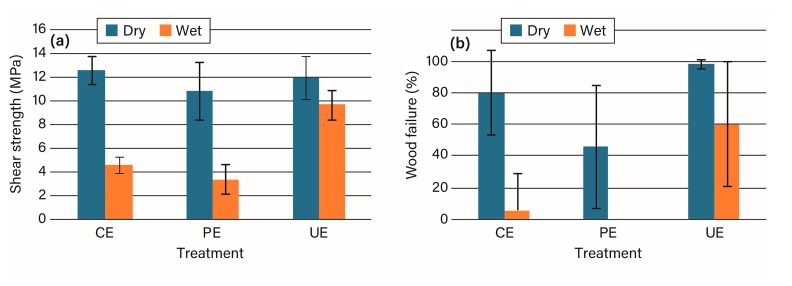
The (**a**) shear strength and (**b**) percentage wood failure for epoxy bonding of unmodified (CE), planed acetylated (PE), and unplaned acetylated (UE) wood.

Epoxy adhesives are generally poor in bonding to wood after water exposure [21]. In the previous study, the epoxy–polyamide adhesive showed a somewhat decreased bond strength and wood failure with acetylated wood in dry tests compared to the unmodified wood and variable strengths and no wood failure in the wet test for any of the wood materials [4]. However, in these experiments, the unplaned acetylated wood provided surprisingly good wet performance compared to the planed acetylated wood and the unmodified wood (Figure 4, Tables S3 and S5). Given the hydrophobic and non-aqueous nature of the epoxy–polyamide, it seems that the fully acetylated surface offers a better bonding surface than the planed acetylated and unmodified wood with their surface hydroxyl groups, which can absorb moisture to disrupt the bond [22]. One limitation is that the epoxy adhesive showed an unusually wide variation in wood failure, but it is not clear why this was observed.

As part of this systematic study with randomized testing, we also bonded samples with the same commercial melamine–formaldehyde used in the prior study, which showed that MF did not bond well to acetylated wood under either dry or wet conditions [4]. However, the specimens showed low strength and low wood failure for the unmodified wood, so the results are not presented here. Examination using visible and fluorescence microscopy showed that the poor results were from overpenetration. Later research showed that if 7.5 wt % of a walnut shell flour filler was added to the MF or a long open assembly time was used, then normal strength and wood failure results were obtained with unmodified wood. Given the randomized nature of the samples in this study, new acetylated samples could not be made and inserted without altering the statistical plan, but the modified MF formulation was used in other studies.

## 4. Discussion

The idea that acetylated wood is too hydrophobic for bonding well with typical wood adhesives is too simplistic for explaining the available adhesive data. For example, it does not explain why the aqueous polar phenolics provide the best bonds to acetylated and non-acetylated wood. The adhesive bonding of acetylated wood is certainly more problematic than that of unmodified wood, and this difference can be dependent upon a number of factors [2,3,4,5,10,18,22,23]:

- As the degree of acetylation increases, the wood absorbs less water.
  ○ This slows the setting of the waterborne adhesives, leading to overpenetration or disruption of polymer formation of those that cure by condensation (water formation).
  ○ This also slows the setting of adhesives that need water for curing, such as isocyanates, which is normally provided by adsorbed water for unmodified wood for curing.
- Acetylation bulks up the wood, allowing for less water absorption to occur upon soaking.
  ○ This reduces the swelling and shrinking of wood as the humidity changes, putting less internal force on the bondline.
  ○ Upon water soaking, the acetylated wood is not plasticized like the unmodified wood; thus, for wet tests, more of the applied load is transferred to the bondline.
  ○ This reduces the free volume for the adhesive to enter into the cell wall.
- Acetylation increases the ability of wood to absorb lower polarity chemicals [20,22].
  ○ This may allow different adhesive components to infiltrate the cell wall compared to the unmodified wood.
- Not all acetylated wood surfaces are the same.
  ○ Method of acetylation may influence the distribution of acetyl groups, and post-treatment steps, such as washing to remove unreacted acetic anhydride and acetic acid, may extract some wood components.
  ○ As shown in this study, planing of the surface alters the surface by increasing the surface hydroxyl content.

One caution is that work was done using one type of acetylation, and not enough work has been done to know the effect of other acetylation conditions on surface properties of the acetylated wood [23]. Additionally, not enough is known about the interaction of each type of adhesive with the wood surface on a cellular level to predict the influence of each factor listed above to explain the performance of each adhesive. Despite certain adhesive formulations or wood type giving acceptable bond performance in some cases, the phenolics (resorcinol–formaldehyde, phenol–formaldehyde, etc.) have most consistently provided strong bond performance in this and other studies [4,8]. The very good bonding with aqueous phenolics is inconsistent with the hypothesis that bonding difficulties of acetylated wood are just a matter of lower polarity of the acetylated wood. With planed surfaces having a higher hydroxyl content, they may be easier to bond than the almost fully acetylated unplanned wood surfaces. In addition, the highly basic phenolics are known to hydrolyze acetyl groups on wood cell wall polymers [24]. It also points out that a waterborne adhesive can lose enough water to acetylated wood and not always overpenetrate the wood, and that these adhesives that give off water by condensation curing can still function properly. However, given the good performance of the phenolics, the poorer performance of melamine–formaldehyde resins as reported in the literature is surprising [4,5,8]. They both fall into the group of in situ polymerized adhesives [9] and are very similar in their chemistry of reacting first with formaldehyde and then condensing to further polymerize. Thus, the most likely explanation seems to be how they interact with the wood. The available data do not provide any evidence that either should appreciably infiltrate into the acetylated wood cell walls [22], although both amino and phenolic resins are well known to infiltrate wood cell walls. It may be due to greater polarity of the melamine with its larger number of amine groups causing failure at the interface rather than the chemistry and mechanics of the polymerization step. Another possibility is that the amino adhesives are more plasticized than the phenolic adhesives and cannot withstand the higher force applied by the acetylated wood under wet conditions. However, methods will need to be developed to sort out the differences between these two adhesive types.

Another type of in situ adhesive is epoxy resin, which has been shown not to provide moisture-durable wood bonds with unmodified wood. It has been proposed that the poor bond durability is caused by the epoxy near the interface not being able to withstand the swelling of the wood [21]. In agreement with the prior study [4], the planed acetylated wood had poorer strengths and wood failure for both dry and wet specimens. However, with the unplaned acetylated wood, the epoxy showed high strength and wood failure for the dry and especially for the wet specimens. The failure of epoxy adhesives with wood has been explained as an inability of the epoxy near the wood surface to accommodate the swelling of the wood [21,25]. The current data seem to indicate that the generation of additional hydroxyl groups by planing causes enough disruption to weaken the epoxy–wood bond under soaking conditions. This did not seem to be true for the unplaned acetylated wood with the epoxy adhesive, where high strength and wood failure were observed. This combination showed good compatibility of the interphase.

The only pre-polymerized adhesive tested in this study was the emulsion polymerized isocyanate. The EPI showed similar reduction in performance with acetylated wood as was observed in prior studies [4,8]. There was no real difference using the planed or unplaned wood in the results in this study. The lack of moisture and moisture adsorption of the wood could interfere with the curing process.

A main problem in bonding to acetylated wood seems to be that the acetylated wood adsorbs less water that can either slow the removal of water from adhesives or not be able to supply as much water to adhesives needed for curing. This could mean that a new formulation that takes these factors into account may provide more durable bonding. The other aspect is that the limited plasticization of the acetylated wood by water means that greater forces are imparted on the bondline at a time when the adhesive may be more plasticized.

## 5. Conclusions

Acetylated wood can be bonded with adhesives that perform as well as or better than the bond performance with unmodified wood. The resorcinol–formaldehyde adhesive formed strong bonds under dry and wet conditions for the unmodified and planed and unplaned acetylated wood. An epoxy adhesive bonded better to the unplanned acetylated and unmodified wood under wet testing and equally well under dry conditions. The emulsion polymer isocyanate had good strength under dry conditions for the acetylated wood. Despite the low moisture level and moisture adsorption of acetylated wood, the acetylated wood puts more force on the bondline because the wood is not plasticized by the water. The poorer performing adhesives may need to be reformulated, or the assembly process modified, to work with acetylated wood. Other adhesives, such as polyurethanes, may have completely different results than shown in this study.

A method that reacts free hydroxyls with trifluoroacetic anhydride followed by X-ray photoelectron spectroscopy yielded data indicating that a planed acetylated surface has 40% of the free hydroxyls in the unmodified wood, whereas an unplaned surface has only 8%. Thus, planing the acetylated wood, as is done for structural products, provides a different surface than would be present for the acetylated wood used in composites. Of the three adhesives tested, only with the epoxy-polyamide did planing make a difference where the unplaned acetylated wood formed a very strong bond under both dry and wet conditions, but planing dramatically reduced the wet strength.

## Figures and Tables

**Table 1:** Relative amount of free surface hydroxyls by trifluoroacetylation of specimens.

Specimen	[CF_3_]/[ΣCarbon]	Free OH Relative to Control
Yellow-poplar	0.13	1.00
Acetylated poplar	0.011	0.09
Acetylated poplar with planing	0.054	0.42

## Supplementary Materials

The supplementary materials are available online at www.mdpi.com/2073-4360/9/12/731/s1, Table S1: Coding for different bonded specimens, Table S2: Mean values of dry shear strengths of all treatments with underlines showing those grouping by the ANOVA analysis, Table S3: Mean values of wet shear strengths of all treatments with underlines showing those grouping by the ANOVA analysis, Table S4: Mean values of dry wood failure of all treatments with underlines showing those grouping by the ANOVA analysis, Table S5: Mean values of wet wood failure of all treatments with underlines showing those grouping by the ANOVA analysis.

## References

[1] Clausen C.A., Ross R.J. (2010). Biodeterioration of Wood. Wood Handbook—Wood as an Engineering Material.

[2] Hill C.A.S. (2006). Wood Modification: Chemcial, Thermal and Other Processes.

[3] Rowell R.M., Rowell R.M. (2013). Chemical modification of wood. Handbook of Wood Chemistry and Wood Composites.

[4] Vick C.B., Rowell R.M. (1990). Adhesive bonding of acetylated wood. Int. J. Adhes. Adhes..

[5] Bongers F., Meijerink T., Lütkemeier B., Lankveld C., Alexander J., Militz H., Lehringer C. (2016). Bonding of acetylated wood. Int. Wood Prod. J..

[6] Rowell R.M., Youngquist J.A., Sachs I.B. (1987). Adhesive bonding of acetylated aspen flakes 1. Surface changes, hydrophobicity, adhesive penetration and strength. Int. J. Adhes. Adhes..

[7] Youngquist J.A., Sachs I.B., Rowell R.M. (1988). Adhesive bonding of acetylated aspen flakes 2. Effects of emulsifiers on phenolic resin bonding. Int. J. Adhes. Adhes..

[8] Vick C.B., Larsson P.C., Mahlberg R.L., Simonson R., Rowell R.M. (1993). Structural bonding of acetylated Scandinavian softwoods for exterior lumber laminates. Int. J. Adhes. Adhes..

[9] Frihart C.R. (2009). Adhesive groups and how they relate to the durability of bonded wood. J. Adhes. Sci. Technol..

[10] Norimoto M., Hon D.N.S., Shiraishi N. (2001). Chemical Modification of Wood. Wood and Cellulose Chemistry.

[11] ASTM International (1998). D 905-98 Standard Test Method for Strength Properties of Adhesives Bonds in Shear by Compression Loading.

[12] ASTM International (2011). D 2559-12AE1 Standard Specification for Adhesives for Structural Laminated Wood Products for Use under Exterior Exposure Conditions.

[13] ASTM International (1999). Standard Practice for Estimating the Percent Wood Failure in Adhesive Joints.

[14] Beecher J.F., Frihart C.R. (2006). X-ray Photoelectron Spectroscopy for Characterization of Wood Surfaces in Adhesion Studies. Wood Adhesives 2005.

[15] Wheeler D.R., Pepper S.V. (1990). X-ray photoelectron and mass spectroscopic study of electron irradiation and thermal stability of polytetrafluoroethylene. J. Vac. Sci. Technol. A.

[16] Qin Z., Gao Q., Zhang S., Li J. (2014). Surface free energy and dynamic wettability of differently machined poplar woods. BioResources.

[17] Larsson P., Mahlberg R., Vick C.B., Simonson R., Rowell R.M., Plackett D.V., Dunningham E.A. Adhesive bonding of acetylated pine and spruce. Proceedings of the Pacific Rim Bio-based Composites Symposium; Chemical Modification of Lignocellulosics.

[18] Everhart D.S., Reilley C.N. (1981). Chemical derivatization in electron spectroscopy for chemical analysis of surface functional groups introduced on low-density polyethylene film. Anal. Chem..

[19] Ibach R.E., Rowell R.M., Lee B.G. Decay protection based on moisture exclusion resulting from chemical modification of wood. Proceedings of the 5th Pacific Rim Bio-Based Composites Symposium.

[20] Obataya E., Shibutani S. (2005). Swelling of acetylated wood in organic solvents. J. Mater. Sci..

[21] Frihart C.R. (2006). Are Epoxy-Wood Bonds Durable Enough?. Wood Adhesives 2005.

[22] Obataya E., Gril J. (2005). Swelling of acetylated wood I. Swelling in organic liquids. J. Wood Sci..

[23] Hill C.A.S. (2011). Wood modification: An update. BioResources.

[24] Yelle D.J., Ralph J. (2016). Characterizing phenol—Formaldehyde adhesive cure chemistry within the wood cell wall. Int. J. Adhes. Adhes..

[25] Frihart C.R., Yelle D.J., Wiedenhoeft A.C. What Does Moisture-Related Durability of Wood Bonds Mean?. Proceedings of the COST E34 Workshop on Enhancing Bobdline Performance.

