# Supplementary material for: Adhesives for Achieving Durable Bonds with Acetylated Wood
Source: Polymers (Basel). 2017 Dec 20;9(12):731. doi: 10.3390/polym9120731 (PMC6418570; doi:10.3390/polym9120731)
Supplement: Supplementary file 1 [file polymers-09-00731-s001.pdf]

polymers

MDPI

Article

Adhesives for Achieving Durable Bonds with Acetylated Wood

Charles R. Frihart [1,*], Rishawn Brandon [2], James F. Beecher [2] and Rebecca E. Ibach [3]

[1] Forest Products Laboratory, Madison, WI; cfrihart@fs.fed.us
[2] Formerly at Forest Products Laboratory, Madison, WI
[3] Forest Products Laboratory, Madison, WI; ribach@fs.fed.us
* Correspondence: cfrihart@fs.fed.us; Tel.: +1-608-231-9208

Supplement: Statistical Analysis of Dry and Wet Sher Samples.

Analyses of variance (ANOVA) were conducted for shear strength and wood failure in both dry and wet conditions for these bonded samples. Mean shear strength values passed the test of normality at the 0.05 probability level; therefore, a parametric ANOVA was conducted using test joint assembly variation to test for treatment effects. Due to wood failure being a bounded determination of 0 to 100, mean percentages of wood failure were not normally distributed; therefore, a non-parametric ANOVA based on median rather than mean values was used to test assembly variation against treatment effects. Parametric ANOVA were also conducted using mean percentages of wood failure, recognizing the data were not normally distributed. Mean rather than median treatment values for wood failure are reported in Tables S4 and S5.

Table S1. Coding for different bonded specimens

Treatment	Description
A	Control Epoxy
B	Control Emulsion Polymer Isocyanate
C	Control Melamine-Formaldehyde
D	Control Resorcinol Formaldehyde
E	Acetylated Planed Epoxy
F	Acetylated Planed Emulsion Polymer Isocyanate
G	Acetylated Planed Melamine–Formaldehyde
H	Acetylated Planed Resorcinol Formaldehyde
I	Acetylated Unplaned Epoxy
J	Acetylated Unplaned Emulsion Polymer Isocyanate
K	Acetylated Unplaned Melamine–Formaldehyde
L	Acetylated Unplaned Resorcinol Formaldehyde

Table S2. Mean values of dry shear strengths of all treatments with underlines showing those grouping by the ANOVA analysis.

Treatment

A	L	I	J	D	B	F	C	G	E	H	K
12.45	12.46	11.65	11.57	12.39	11.8	11.6	12.07	11.36	11.07	10.12	8.48

Epoxy

A	I	E
12.45	11.65	11.07

Emulsion Polymer-Isocyanate

J	B	F
11.57	11.8	11.6

Melamine

C	G	K
12.07	11.36	8.48

Resorcinol

L	D	H
12.46	12.39	10.12

The data in Table S2 shows that when tested under dry conditions all the adhesives bonds provided good shear strength. In the case H where the bond is lower in strength is most likely due to the variability in the wood since the wood failure is high (Table S3).. Other cases of low strength also had low wood failure.

Table S3. Mean values of wet shear strengths of all treatments with underlines showing those grouping by the ANOVA analysis.

Treatment

L	I	H	G	D	B	K	C	A	E	J	F
9.40	9.77	9.55	7.77	5.58	5.55	6.04	4.94	4.47	2.98	3.33	3.03

Epoxy

I	A	E
9.77	4.47	2.98

Emulsion Polymer-Isocyanate

B	J	F
5.55	3.33	3.03

Melamine

G	K	C
7.77	6.04	4.94

Resorcinol

L	H	D
9.4	9.55	5.58

Table S3 shows that different wood modifications and adhesives gave a range of results that can be grouped in different ways, but four combinations provide distinctly better strength. In comparing the data in Tables S2 and S3, the resorcinol–formaldehyde retains its strength under wet conditions for the acetylated wood, but the weaker measured bond strength for the control is caused by water

plasticization weakening the wood. For the epoxy its bond strength was only retained under the wet conditions for the unplanned acetylated wood. This wood with the fewest hydroxyls on the surface should swell the least near the interface. The high strength with the melamine–formaldehyde with planed acetylated wood is likely due to the hydroxyl groups to interact with on the surface, but the overpenetration of the MF was limited by the poor absorption into the wood. For the emulsion polymer isocyanate, the control wood did not overlap those with the two acetylated wood.

Table S4. Mean values of dry wood failure of all treatments with underlines showing those grouping by the ANOVA analysis.

Treatment

B	D	I	L	H	F	A	J	E	C	G	K
100	100	100	100	100	100	90	92.5	30	30	0	0

Epoxy

I	A	E
100	90	30

Emulsion Polymer-Isocyanate

B	F	J
100	100	92.5

Melamine

C	G	K
30	0	0

Resorcinol

D	L	H
100	100	100

In Figure S4, the high wood failures for all adhesives, but one epoxy and all the melamine-formaldehyde bonds was in some ways surprising. Normally the melamine-formaldehyde forms strong bonds to the unmodified wood. As stated in the article this is why we excluded MF from the main text since the adhesive overpenetrated to form a starved bond which was proven by later experimentation. The distinct group for 100% wood failure is due to a low variance at the limit in this bounded measurement. The wide variation of results for the epoxy is illustrated by the values of 30 and 90 being overlapped.

Table S5. Mean values of wet wood failure of all treatments with underlines showing those grouping by the ANOVA analysis.

Treatment

D	B	H	L	I	C	A	G	E	F	K	J
100	100	100	100	60	20	0	0	0	0	0	0

Epoxy

I	A	E
60	0	0

Emulsion Polymer-Isocyanate

B	F	J
100	0	0

Melamine

C	G	K
20	0	0

Resorcinol

D	H	L
100	100	100

In Table S5, the data for the melamine-formaldehyde bonding to the unmodified wood under these wet conditions was surprising and it was later shown to be a poor formulation that overpenetrated the wood and caused a starved bondline. The high wood failure for the phenol–formaldehyde is consistent with prior results. Since unplanned acetylated wood has not been tested before, the high wood failure is very unusual for an epoxy and wood under wet conditions.

© 2017 by the authors. Submitted for possible open access publication under the terms and conditions of the Creative Commons Attribution (CC BY) license (http://creativecommons.org/licenses/by/4.0/).
